# An engine for global plant diversity: highest evolutionary turnover and emigration in the American tropics

**DOI:** 10.3389/fgene.2015.00130

**Published:** 2015-04-08

**Authors:** Alexandre Antonelli, Alexander Zizka, Daniele Silvestro, Ruud Scharn, Borja Cascales-Miñana, Christine D. Bacon

**Affiliations:** ^1^Department of Biological and Environmental Sciences, University of GothenburgGöteborg, Sweden; ^2^Gothenburg Botanical GardenGöteborg, Sweden; ^3^Department of Ecology and Evolution, University of LausanneLausanne, Switzerland; ^4^Laboratoire de Paléobiogéologie, Paléobotanique, Paléopalynologie, Département de Géologie, Université de LiègeLiège, Belgium; ^5^Laboratório de Biología Molecular (CINBIN), Department of Biology, Universidad Industrial de SantanderBucaramanga, Colombia

**Keywords:** angiosperms, biogeography, diversification rates, latitudinal diversity gradient, out-of-the-tropics, phylogenetics, tropical biodiversity

## Abstract

Understanding the processes that have generated the latitudinal biodiversity gradient and the continental differences in tropical biodiversity remains a major goal of evolutionary biology. Here we estimate the timing and direction of range shifts of extant flowering plants (angiosperms) between tropical and non-tropical zones, and into and out of the major tropical regions of the world. We then calculate rates of speciation and extinction taking into account incomplete taxonomic sampling. We use a recently published fossil calibrated phylogeny and apply novel bioinformatic tools to code species into user-defined polygons. We reconstruct biogeographic history using stochastic character mapping to compute relative numbers of range shifts in proportion to the number of available lineages through time. Our results, based on the analysis of c. 22,600 species and c. 20 million geo-referenced occurrence records, show no significant differences between the speciation and extinction of tropical and non-tropical angiosperms. This suggests that at least in plants, the latitudinal biodiversity gradient primarily derives from other factors than differential rates of diversification. In contrast, the outstanding species richness found today in the American tropics (the Neotropics), as compared to tropical Africa and tropical Asia, is associated with significantly higher speciation and extinction rates. This suggests an exceedingly rapid evolutionary turnover, i.e., Neotropical species being formed and replaced by one another at unparalleled rates. In addition, tropical America stands out from other continents by having “pumped out” more species than it received through most of the last 66 million years. These results imply that the Neotropics have acted as an engine for global plant diversity.

## Introduction

The world's biodiversity is unevenly distributed, and most species are found in the tropical regions of Asia (including Australasia), Africa, and the Americas. Understanding the underlying causes for the latitudinal biodiversity gradient—the decrease of taxonomic diversity away from the equator—has fostered extensive and integrative research, and its formation still constitutes a matter of debate in evolutionary biology and biogeography (see e.g., Pianka, 1966; Hillebrand, 2004; Jablonski et al., 2006; Wiens et al., 2006; Brown, 2014; Huang et al., 2014; Kerkhoff et al., 2014; Mannion et al., 2014; Rolland et al., 2014).

There are three primary explanations for the latitudinal biodiversity gradient, which are not mutually exclusive. Often referred to as the museum hypothesis (Stebbins, 1974), one view is that there has been a longer period of time for the accumulation of diversity in the tropics because most of the Earth was essentially tropical until the Eocene–Oligocene boundary c. 34 million of years ago (Ma; Zachos et al., 2008). In contrast to the focus on geological and evolutionary time, it has also been proposed that higher tropical biodiversity could be caused by higher net diversification rates in tropical vs. temperate zones (Mittelbach et al., 2007), i.e., either due to high speciation, low extinction, or some combination of both. Why such rates would be different is in itself a matter of further debate, with a key role being attributed to kinetics (Brown, 2014). More recently it has been suggested that it is the inability of tropical lineages to disperse, survive, and diversify out of the tropics that drives the latitudinal biodiversity gradient, due to intrinsic eco-physiological constraints (niche conservatism; Kerkhoff et al., 2014).

A second striking feature of tropical biodiversity, besides being consistently higher than in non-tropical regions, is its uneven distribution among the three tropical regions of the world. For instance, it has been suggested that the American tropics (the Neotropics) comprise more species of seed plants than tropical Africa and tropical Asia together, with similar patterns for other organismal groups such as amphibians, mammals, birds, nymphalid butterflies, and reptiles (Govaerts, 2001; Antonelli, 2008; Antonelli and Sanmartín, 2011 and references therein). The underlying causes for these inter-continental differences are poorly understood, and could be analogous to those determining the latitudinal biodiversity gradient. In addition, differences in area and biome sizes, environmental and soil heterogeneity, climatic history, biological exploration, and digitalization of natural history collections (amongst others) could also play important roles.

Evaluating the validity and relative roles of the factors driving these fundamental biodiversity differences requires combining evidence from several sources and disciplines, such as palaeontology, ecology and molecular phylogenetics. Among these, two main components stand out as essential in this pursuit: understanding species diversification (i.e., the interplay between speciation and extinction) and the geographic history of lineages. In this study we explore these two components at a global and continental scale. We focus on the Cenozoic history (i.e., the last 66 Ma) of flowering plants (angiosperms), which form the dominant structure of tropical and temperate ecosystems. We ask two overarching questions:

1. Have the tropics as a whole, and each tropical region separately, been mainly a sink or a source of angiosperm diversity?
  More specifically, did range shifts (including trans-oceanic dispersals) between tropical and non-tropical zones, and into and out of each tropical region, occur in both directions at a roughly constant pace throughout the Cenozoic, or were there phases of markedly different range shift rates and directionality?
2. Is high diversity correlated with high speciation and/or low extinction?
  More specifically, were there significant differences in speciation and extinction rates between tropical and non-tropical zones, and among tropical regions? In such case, are the most species rich regions also those with highest speciation and/or lowest extinction?

To address these questions, we calculate and compare rates of speciation and extinction between tropical and non-tropical zones and among the world's three tropical regions (in Africa, Asia, and the Americas), and we infer the timing and direction of range shifts into and out of each tropical region.

## Material and methods

### Data compilation

Fossils, molecular phylogenies, and species occurrences constitute diverse data sources that, taken together, can be used to infer diversity trends through time and space. Here we explore the feasibility of using both neontological and palaeontological data for addressing the questions outlined in this study.

#### Fossils

**Figure 1:**
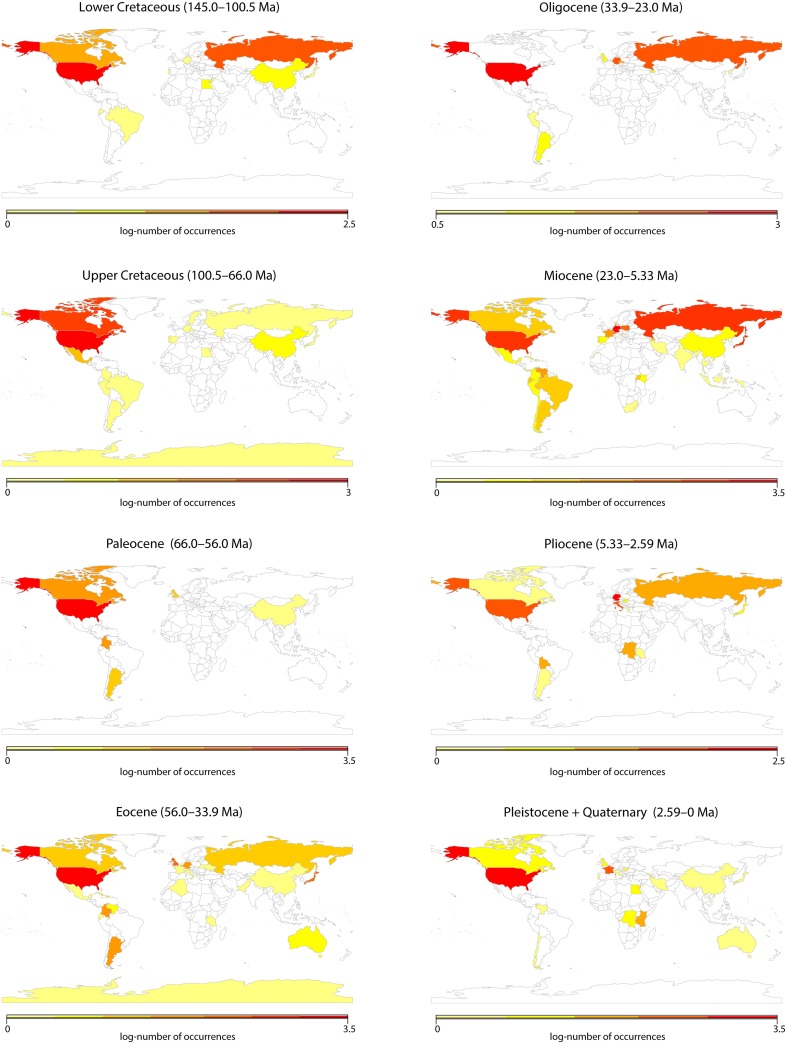
**Visualization of a global data set of angiosperm macrofossil occurrences downloaded from the Paleobiology database as described by Silvestro et al. (2015)**. The data set included 9665 records, representing a total of 297 fossil taxa identified to the genus level. In this figure, all records were subdivided by country and time period, according to the Geological Time Scale of Gradstein et al. (2012).

We explored whether fossils could be used to infer diversity trends through time, as has been recently demonstrated for fossil rich clades such as mammals (Silvestro et al., 2014). For this we assessed a global data set of angiosperm macrofossil occurrences originally downloaded from the Paleobiology database (https://www.paleobiodb.org) as described by Silvestro et al. (2015). The data set included 9,665 records, representing a total of 297 fossil taxa identified to the genus level; identifications below the generic level were grouped by genus. To investigate potential biases in the data, all records were subdivided by country and time period (from the Lower Cretaceous to today), according to the Geological Time Scale of Gradstein et al. (2012). Unfortunately, a visual inspection of the data (Figure 1) showed severe spatial and temporal biases. These biases precluded any sensible analyses of diversity changes in tropical regions, and we were therefore forced to rely on species distribution and molecular data alone.

#### Species occurrences

We downloaded all geo-referenced (i.e., provided with a longitude and latitude) species occurrences of angiosperms available at the Global Biodiversity Information Facility (GBIF, http://www.gbif.org; downloaded in June 2014). Records flagged to contain “known coordinate issues” were excluded prior to the download. One record per location per species was retained. We then applied basic data cleaning steps on the full data set (c. 40 gigabytes) for identifying and excluding obviously erroneous data points, such as records with non-numeric coordinates or missing species names, records with identical latitude and longitude, and latitudes or longitudes equal to zero (which we considered to have been left in blank during data entry). For these steps we used a modified version of the scripts by Zanne et al. (2014) implemented in R (R Core Team, 2014).

#### Geographic assignments

**Figure 2:**
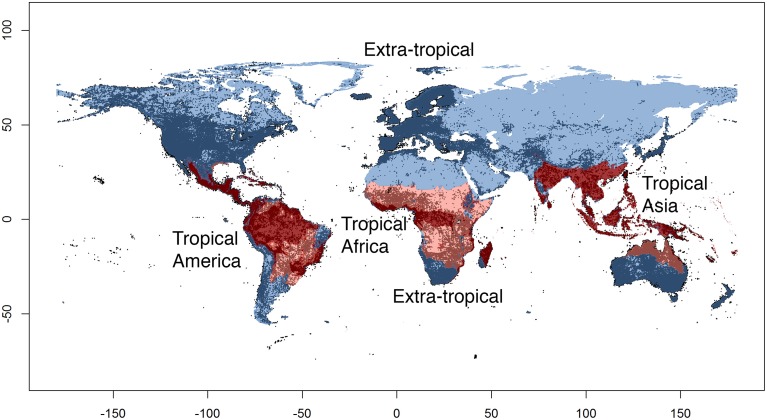
**The four regions (operational units) used in this study**. Tropical regions are shown in red (dark red: rain forests, light red: savannas), non-tropical regions are shown in blue. Dots indicate species occurrence records of angiosperms (c. 20 million) used in this study and obtained from the Global Biodiversity Information Facility (GBIF).

We coded each species for its presence and absence in four large regions or operational units (Figure 2): tropical America (the Neotropics), tropical Africa (the Afrotropics), tropical Asia (including Australasia), and all other (non-tropical) regions combined. We delimited those regions by following the same boundaries for biomes and ecoregions as adopted by the World Wide Fund for Nature (WWF), as described in Olson et al. (2001). We considered the following ecoregions as forming together the tropical region: “Tropical and Subtropical Moist Broadleaf Forests,” “Tropical and Subtropical Dry Broadleaf Forests,” “Tropical and Subtropical Coniferous Forests” and “Tropical and Subtropical Grasslands, Savannas, and Shrublands.” All other ecoregions were merged to form our “non-tropical” region. We classified “Flooded Grasslands and Shrublands” as tropical or non-tropical depending on the surrounding biome and geographic position. We acknowledge that the WWF biome and ecoregion classification is to some extent arbitrary and based on expert opinion, rather than directly data derived (Vilhena and Antonelli, 2014). However, we consider that the level of accuracy of this classification is adequate for the purposes of this study, and superior to a classification based solely on latitudinal limits or a purely climatic classification without proper consideration of biotic components (Kottek et al., 2006).

For each continent, all polygons for biomes classified as “tropical” were merged into a single polygon, and the same was done for all “non-tropical” biomes, which were merged into a single multi-polygon comprising areas in both the southern and the northern hemisphere. This means that each tropical region comprised e.g., both rainforests and savannas, but excluded very dry areas (such as the Sahara in Africa, the Caatinga in South America and parts of the Deccan plateau in India) as well as the coldest habitats (e.g., high altitude areas in the South American Andes and along the African Great Rift Valley) located within the tropical belt (between c. 23° north and c. 23° south). Although smaller operational units would have been interesting from a biological perspective, e.g., separating rain forests and savannas, it would inevitably incur a considerable loss of data and statistical power for the subsequent analyses. We utilized the software package SpeciesGeoCoder v.1.0 (Töpel et al., 2014) to code species into operational units. The resulting polygons can be retrieved from the authors upon request.

To further identify potential biases caused by erroneous geo-references (e.g., due to wrong coordinates or species identifications), we applied a set of arbitrary thresholds in order for a species to be coded as “present” in a certain operational unit. Three filters were defined, with increasingly more strict criteria, as outlined in Table 1. We implemented functions and scripts to carry out this data filtering in R (scripts available from the authors).

**Table 1:** **Automated criteria for recording presence of each species in each operational unit defined in Figure 2, departing from raw GBIF species occurrence data**.

**Filter**	**Minimum # records**	**Minimum % records**	**Presence on more than one polygon**
None	1	–	Allowed
1	3	–	Allowed
2	3	10	Allowed
3	3	10	Not allowed^*^

* *Under this filter, presence was only coded in the region with the highest number of records*.

There was no major loss of occurrence records by going from Filter 1 to the more conservative Filter 2 (see Results below). We therefore chose to perform our analyses on range transitions on the data set generated under Filter 2, and the diversification rate analyses using the Filter 3 data set, due to the fact that the method we employed cannot handle widespread taxa (see below).

#### Molecular phylogeny

We chose to work with a single dated tree rather than performing a meta-analysis of individual trees (e.g., Jansson et al., 2013, so that divergence times among clades would be more directly comparable with each other. We therefore used the recent fossil-calibrated molecular phylogeny of angiosperms from Zanne et al. (2014), with 30,535 species. The phylogeny was based on data from seven gene regions and families and orders were constrained to the APG III classification system (Bremer et al., 2009). To evaluate whether the level of taxonomic representation was consistent among regions, which could otherwise bias our subsequent analyses, we calculated the ratio between the number of species sampled in the phylogeny and the total number of species recorded in each of the four regions in the GBIF database.

#### Tropical conservatism

We tested whether species in each of the regions defined (Figure 2) were clustered in the angiosperm phylogeny (i.e., showed strong phylogenetic signal) using Bayesian Tip-Significance testing implemented in the software BaTS v. 1.0 (Parker et al., 2008). We compared the observed distribution of states in the reference phylogeny against 100 randomized replicates, which were used to compute 95% credible intervals of trait distributions.

#### Range shifts through time

We used the region-coded, dated phylogeny of angiosperms to estimate the timing and directionality of range shifts between tropical and non-tropical lineages, and among the three tropical regions of the world. Since our analyses focused on the Cenozoic, when the three tropical continents were already widely separated by oceans (Mcloughlin, 2001), these events should include both trans-oceanic dispersals as well as range expansions over continuous land between the tropical and non-tropical zone.

We used stochastic character mapping (Huelsenbeck et al., 2003) to reconstruct histories of shifts across biogeographic regions (e.g., Clark et al., 2008). We calculated the relative number of transitions through time (Silvestro, 2012; Fernández-Mendoza and Printzen, 2013) as the absolute number of transitions divided by the number of nodes in 5 million year time bins. We did this to account for the fact that even under a simple birth model of speciation the number of lineages in a phylogeny tends to increase exponentially, therefore increasing the possibility of range shifts to occur toward the present (Silvestro, 2012). Credible intervals around the relative number of transitions through time were obtained by simulating 100 stochastic histories of geographic range evolution. We optimized the original scripts implementing this method and implemented them in R using phytools (Revell, 2012) to perform stochastic mapping (new scripts available from the authors).

#### Diversification rates

We calculated rates of speciation (λ) and extinction (μ) for each tropical region separately, as well as for tropical and non-tropical species. For these analyses we used the Multiple State Speciation and Extinction method (MuSSE) as implemented in diversitree (Fitzjohn, 2012). We analyzed 17 subclades separately (Table 2), which we chose to correspond to plant orders. This division was necessary due to computational limitations in analysing the full tree under this method, but also carried the advantage of creating a sample of rate estimates across different angiosperm clades. We did not explore the effect of splitting the angiosperm tree into different numbers of subclades or along different branches, since there would be an almost endless number of possible combinations. We accounted for varying levels of taxonomic sampling in the phylogeny by calculating the sampling fraction of each order.

**Table 2:** **Proportion of species included in the phylogeny for each plant order analyzed**.

**Order**	**Total # spp**	**# In diversification analyses**	**Sampling in tropics**	**Sampling outside tropics**
Apiales	3114	478	0.07	0.17
Asparagales	13956	1601	0.08	0.13
Asterales	19213	1958	0.03	0.12
Brassicales	2945	497	0.07	0.17
Caryophyllales	7992	945	0.07	0.11
Ericales	8305	1233	0.09	0.18
Fabales	15049	2050	0.09	0.13
Gentianales	11583	1052	0.08	0.07
Lamiales	14813	1397	0.04	0.12
Malpighiales	10882	1219	0.09	0.09
Malvales	4398	446	0.07	0.10
Myrtales	8439	825	0.06	0.12
Poales	13872	2190	0.06	0.18
Ranunculales	2776	468	0.07	0.18
Rosales	6396	852	0.09	0.13
Sapindales	4991	667	0.10	0.12
Solanales	3298	512	0.10	0.14

*Species numbers follow those in the GBIF Backbone Taxonomy. (Sampling in tropics = number of species in the diversification analyses classified as tropical divided by the total number of species classified as tropical; Sampling outside the tropics = number of species in the diversification analyses classified as outside the tropics divided by the total number of species classified as outside the tropics)*.

We compared the significance of results from the diversification analyses using Analysis of Variance (ANOVA), and then applied the Tukey's honest significant difference (HSD) test in order to identify outstanding values. To account for intrinsic differences among plant orders, we normalized the rates of speciation and extinction for each order over all regions. This was done by dividing each rate by the sum of the rates in all regions analyzed. In all analyses, we used mean values of rates.

## Results

### Data compilation

**Figure 3:**
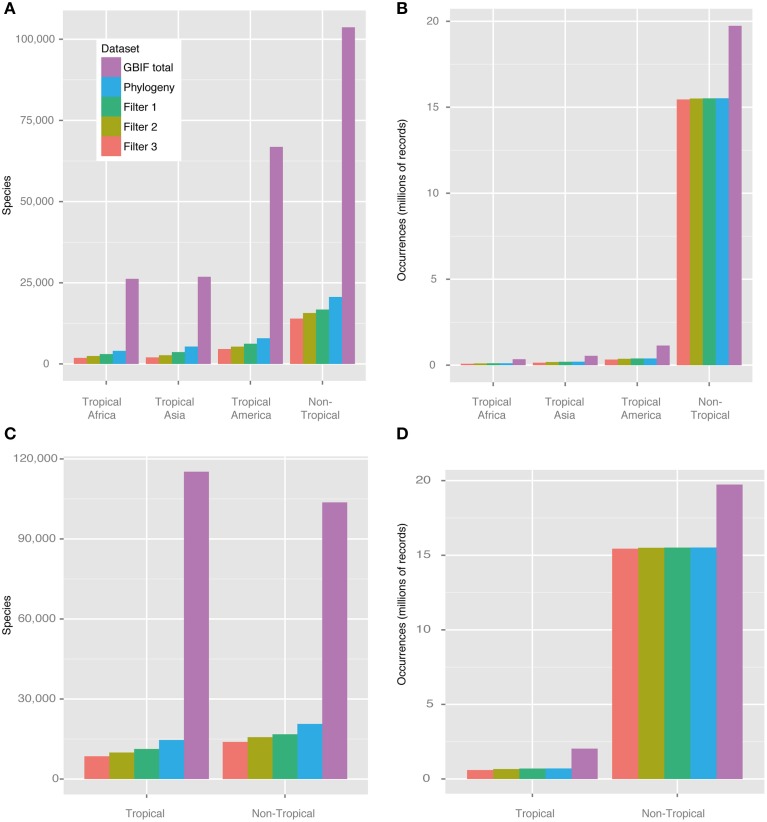
**Number of angiosperm species and occurrences in the four regions defined in this study**. The bars show the influence of different cleaning steps on the data set (see also Table 1). **(A)** Number of species per dataset and geographic region, **(B)** number of occurrence points per dataset and geographic region, **(C)** number of species per dataset and geographic region (Tropical vs. Non-Tropical), **(D)** number of occurrence records per dataset and geographic region (Tropical vs. Non-Tropical). Purple: GBIF download; blue: species that are included (and could be matched) in the phylogeny; dark green: Filter 1 (minimum 3 occurrences to be coded as present in a given region); light green: Filter 2 (additionally 10% of all occurrences per species needed to be coded as present); orange: Filter 3 (additionally widespread species restricted to one region). The Filter 2 data set was used for all analyses except for MuSSE and BaTS.

Figure 3 shows the number of species and occurrences coded into each of the regions defined, the number of those that were also present in the phylogeny, and the influence of each filter applied. The raw data set of species occurrence points (after applying the basic cleaning steps described above) comprised a total of 24,908,478 records pertaining to 188,655 species (purple bars, Figure 3). Many species could not be matched between the species occurrence data set and the molecular phylogeny used, due to taxonomic issues that could not be easily solved (e.g., synonymisation and different taxonomic circumscriptions), and the fact that numerous species did not occur in both data sets. Despite these issues, a total of 27,585 species could be fully matched between the molecular phylogeny and the occurrence data set, representing 14.6% of the total number of currently accepted species of angiosperms (273,174 species, according to http://www.theplantlist.org; accessed September 2014). The data set generated under Filter 2, used for all analyses except MuSSE and BaTS, comprised a total of c. 20 million occurrence points and between c. 500 to 6600 species per region (Figure 3).

The proportion between species with geo-references and species in the phylogeny ranged from c. 8 to 15% among regions (Table 3). All tropical regions were similarly represented in the phylogeny, with only 2% difference between the best sampled tropical region (tropical Asia) and the least sampled one (tropical America). Non-tropical regions were better sampled phylogenetically than tropical ones (15% vs. 9%, respectively).

**Table 3:** **Number of species recorded in each of the regions defined for the analyses (for which georeferenced data were available from GBIF), number of those species that could be included in the range shift analysis (after applying Data Filter 2; see Table 1 and Figure 3), and their sampling fraction**.

**Region**	**Total # spp (GBIF)**	**# In range shift analyses**	**Sampling fraction**
African tropics	26194	2460	0.09
American tropics	66844	5342	0.08
Asian tropics	26854	2686	0.10
Total tropics	115196	9913	0.09
Non tropical	103682	15666	0.15

### Phylogeny-based analyses

**Figure 4:**
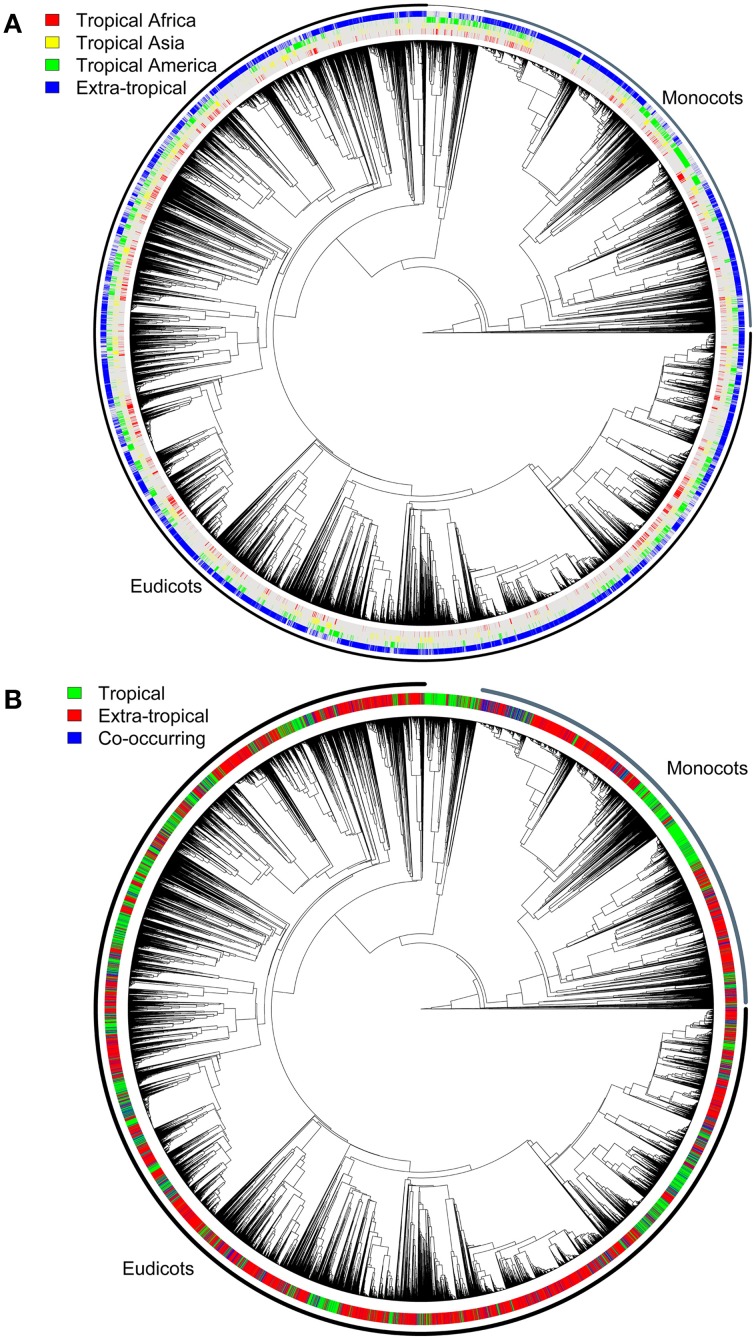
**Angiosperm phylogeny used for the range shift and diversification analyses, pruned from Zanne et al. (2014)**. The tree contains c. 22,600 terminal species and shows **(A)** the codification into each one of the continental-level regions defined in Figure 2, and **(B)** the codification of all species as tropical or non-tropical. Species in each of the regions defined are highly clustered phylogenetically according to Bayesian Tip-Significance testing (*p* < 0.001).

Figure 4A shows the angiosperm phylogeny and the coding of each species as occurring in each of the four regions defined, whereas Figure 4B shows the coding in tropical and non-tropical regions. The Bayesian Tip-Significance testing indicated that species in all regions (Figure 2) are highly clustered phylogenetically (*p*<0.001 for all three statistical tests implemented in BaTS: parsimony score, association index and maximum exclusive single-state clade).

**Figure 5:**
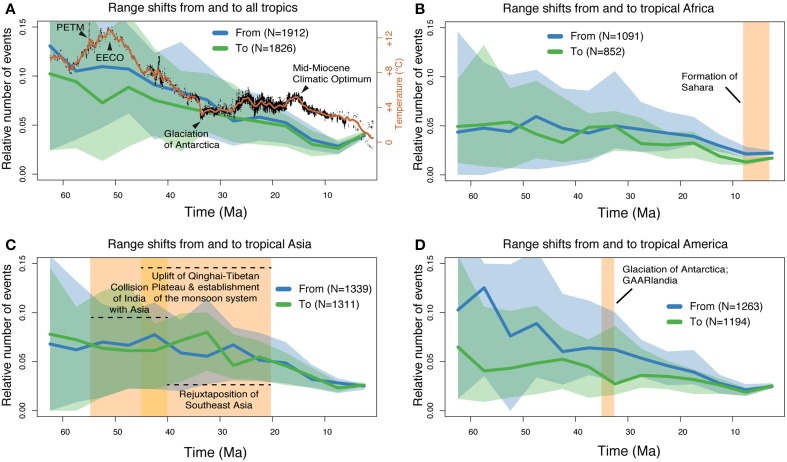
**Results from the range shift analyses using stochastic mapping**. The plots show the number of range shift events (including long-distance dispersals and continuous range expansions) through time, relative to the available number of lineages (see Methods for details). **(A)** Inferred number of range shift events into and out of the tropical zone; **(B–D)** rate estimations for tropical Africa, Asia, and America, respectively. The 95% confidence intervals are shown as shaded areas in all plots. In **(A)**, a global mean temperature curve (Hansen et al., 2008) is shown in red for comparison. The yellow boxes in the other figures are shown as references for the discussion. EECO, Early Eocene Climatic Optimum; PETM, Paleocene-Eocene Thermal Maximum; GAARlandia, Greater Antilles and Aves Ridge landbridge.

The results from the range shift analyses are summarized in Figure 5. Confidence intervals of range shift rates were generally large and mostly overlapping, but the width of their ranges decreased toward the present. During most of the Cenozoic, mean emigration rates (out of the tropics) were slightly higher or very similar to migration into the tropics (Figure 5A). From c. 58 to c. 44 Ma, immigration into the tropics showed a small decrease. Both tropical Africa (Figure 5B) and tropical Asia (Figure 5C) showed similar mean rates of immigration and emigration through time, except for some fluctuations (especially in Asia, prior to c. 25 Ma). In contrast, there was a consistently higher rate of emigration from tropical America (Figure 5D). These rates only reached equilibrium c. 14 Ma.

**Figure 6:**
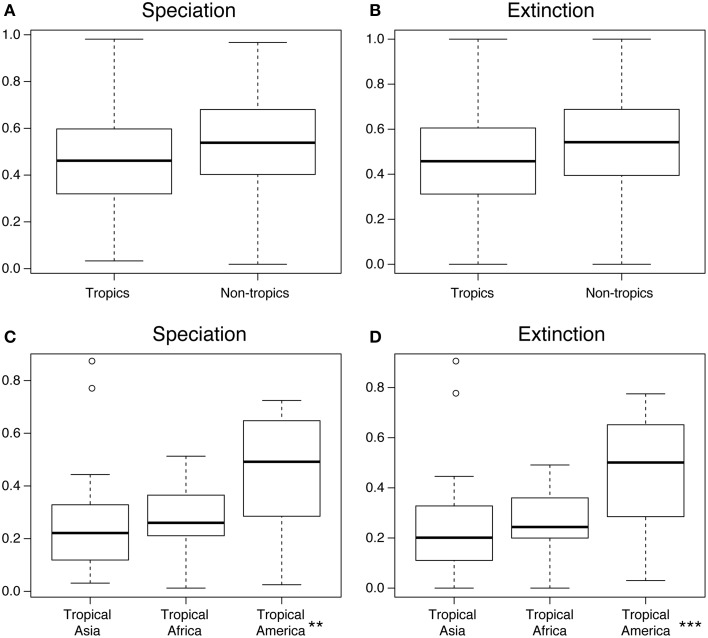
**Results from the diversification rate analyses under the MuSSE model**. **(A)** Speciation rates per geographic region (tropical vs. non-tropical); **(B)** Extinction rates per geographic region (tropical vs. non-tropical); **(C)** Speciation rates for the three tropic regions; **(D)** Extinction rates for the three tropic regions. All results are normalized against each other. Each data point represents an angiosperm plant order (Table 2). Boxes indicate the interquartile range (IQ) of all estimates, with the median shown as a horizontal line and the whiskers indicating data range outside the quantiles. ^**^ and ^***^ denote significant differences (*p* < 0.05 and *p* < 0.001, respectively; ANOVA). See Methods for details.

The region-specific rates of speciation and extinction inferred using the MuSSE model are shown in Figure 6, calculated under the sampling fractions for each order indicated in Table 2. Individual estimates are reported in Supplementary Table S1, and significance values in each set of comparisons are summarized in Table 4.

Table 4**Variables and statistical tests based on the MuSSE analyses of the molecular phylogeny of angiosperms**.

**Table:** 

**Variable and sample for comparison**	***p*-value**	**n (orders/region)**
Speciation: tropical vs non-tropical	0.521	17
Extinction: tropical vs non-tropical	0.516	17
Speciation: among tropical regions	0.0107	17
Extinction: among tropical regions	0.00839	17

**Table:** 

**TUKEY HSD FOR ANOVA AMONG TROPICAL REGIONS**				
**Rate**	**America vs. Africa**	**America vs. Asia**	**Africa vs. Asia**	**Outstanding region**
Speciation	0,0209	0,0256	0,9965	American tropics
Extinction	0,0175	0,0205	0,9979	American tropics

*Significant values at 95% confidence levels are underscored*.

The median values of both speciation and extinction rates were higher in non-tropical than in tropical zones, but these estimates showed large overlap in their confidence intervals and are not statistically different (Figures 6A,B). In contrast, both the speciation and the extinction rates estimated for tropical America were significantly higher than those estimates for tropical Africa and tropical Asia (Figures 6C,D, *p* < 0.05 for speciation, and *p* < 0.001 for extinction).

## Discussion

### The geographic history of tropical angiosperms

Our analyses of historical range shift events (Figure 5) reveal some interesting patterns. During the first half of the Cenozoic (from 66 until c. 30 Ma), our results indicate that most range shifts took place out of the tropics. This result corroborates a recent meta-analysis of 111 dated phylogenies, including seven clades of angiosperms (Jansson et al., 2013), and also reflects the directionality observed from the fossil record of marine bivalves for the last 11 Ma (Jablonski et al., 2006, 2013), Overall, range shifts appear poorly associated in time with climate, approximated through a mean global temperature curve (Figure 5A). Some correspondence may however include a c. 30% decrease in range shifts into the world's tropics during the highest temperature levels of the Cenozoic, around the Early Eocene Climatic Optimum c. 52 Ma (Zachos et al., 2008). An additional overall decrease is observed coinciding with the Mid-Miocene Climatic Optimum c. 15 Ma. Why global warming would have influenced range shifts among tropical and non-tropical regions as observed here is puzzling, and may reflect large-scale but poorly understood vegetational changes. We also note that range shifts into and out of the tropics reached an equilibrium only a few million years after the Eocene-Oligocene transition, a global cooling event associated with the gradual glaciation of Antarctica (Zachos et al., 2008).

Range shifts into and out of tropical Africa (Figure 5B) occurred in both directions at about the same rate, and showed the least fluctuations among the three tropical regions analyzed. The initial formation of the Sahara c. 7 Ma (Zhang et al., 2014) did not seem to leave a considerable footprint on these rates.

Range shifts into and out of tropical Asia (Figure 5C) were fairly similar and exhibited most fluctuations prior to c. 23 Ma. Major events in that period include the collision of India with Asia c. 55–45 Ma, the uplift of the Qianghai-Tibetan Plateau c. 45–20 Ma, and the establishment of the monsoon system in Southeast Asia c. 35–20 Ma (Favre et al., 2014). The “out-of-India” hypothesis postulates that a number of African-derived organisms, including both animals (Bossuyt and Milinkovitch, 2001) and plants (Conti et al., 2002), rafted on the Indian subcontinent and dispersed into Asia after the collision of these landmasses. This dispersal route has received support from the molecular analyses of several taxa (Karanth, 2006). We note a temporal correlation between the initial collision (c. 55 Ma) and the shift from tropical Asia being mainly a sink of lineages to it becoming a net source of angiosperm diversity. Another major event in the Cenozoic is the geological rejuxtaposition of Southeast Asia, which created a stepping-stone route between Oceania and Asia from c. 40 Ma (Hall, 2009). This event might be reflected in our results by the increase of lineages entering tropical Asia around that time, leading again to a net input of non-tropical lineages into tropical Asia.

Range shifts out of tropical America were consistently more frequent that those entering it, throughout most of the Cenozoic (from c. 65 to 15 Ma; Figure 6D). A remarkable peak in emigration shifts was estimated at c. 57 Ma, which was simultaneously associated with a modest decrease in immigration events. These results imply a c. 3 times higher rate of lineages leaving the Neotropics than shifts in the opposite direction. We note that this peak corresponds closely in time (allowing for the uncertainties in molecular dating) to the Paleocene-Eocene Thermal Maximum (PETM; Figure 5A). This was a short-lived (c. 10,000 years) event which took place c. 56.3 Ma and was characterized by mean global temperatures reaching above 12°C from today's level (Zachos et al., 2008). Evidence from the fossil record show that considerable changes occurred at the PETM in Neotropical rainforests, with rapid origination of new taxa and changes in vegetation composition due to range shifts and local extirpations (Jaramillo et al., 2010). It seems therefore reasonable to suggest that newly speciated taxa might, at least in part, account for the inferred peak.

The high rate of range shifts out of the Neotropics is particularly noteworthy in comparison to the other tropical regions, where we did not find this difference between immigration and emigration. Thus, our results suggest that the Neotropics have functioned as a “species pump” for the rest of the world during the first 50 million years of the Cenozoic, but in particular during the Paleocene and early Eocene. The reasons for this require further investigation, but reflect the patterns observed in marine bivalves in which clades with higher diversification were the most likely to expand out of the tropics (Jablonski et al., 2013).

A second event of potential significance for range shifts in the Neotropics was the establishment of a stepping-stone land bridge reducing the gap between North and South America, known as the Greater Antilles and Aves Ridge or GAARlandia (Iturralde-Vinent and Macphee, 1999; Pennington and Dick, 2004). The existence and role of the GAARlandia in facilitating dispersals remains controversial (Ali, 2012), but the hypothesis has gained recent support in phylogeographic analyses of several animal taxa, including spiders (Crews and Gillespie, 2010), amphibians (Alonso et al., 2012) and cichlids (Říčan et al., 2013). We did not detect any definite signal of the GAARlandia in our estimation of range shifts for angiosperms, except perhaps for a slow *decrease* in shifts entering the Neotropics (which, if confirmed, could also be linked to the global temperature decline at Eocene/Oligocene transition).

### Building up tropical biodiversity

Our phylogeny-based estimates of speciation and extinction rates (Figure 6) showed that angiosperms in tropical regions both speciated and went extinct at lower rates than in temperate regions, although this difference was not significant (*p* > 0.05; Table 4). This result reflects the lack of conclusive evidence on this issue. Several studies have suggested higher rates of diversification (defined as speciation minus extinction) in the tropics (Mittelbach et al., 2007), including amphibians (Pyron and Wiens, 2013), mammals (Rolland et al., 2014), and squamate reptiles (Pyron, 2014). Others have found temperate regions to have higher diversification rates, based on the analysis of birds and mammals (e.g., Weir and Schluter, 2007). An analysis of bird diversification showed yet a third pattern, where the major differences in diversification rates were between the western and eastern hemispheres, rather than between tropical and temperate zones (Jetz et al., 2012). Our results are similar to those obtained by Jansson et al., (2013), who found no significant differences in the net diversification between tropical and temperate sister lineages. Overall, our results suggest that the higher diversity of angiosperms in tropical compared to non-tropical regions is not primarily dependent on higher speciation and/or lower extinction in the tropics.

In contrast, our results show significantly different rates of speciation and extinction amongst the tropical regions of the world (Figures 6C,D). Neotropical angiosperms speciated on average about 2–2.5 times faster than angiosperms in tropical Asia and tropical Africa. However, they also went extinct about 2–2.5 times faster than in tropical Asia. These high rates of speciation and extinction in the Neotropics indicate a rapid evolutionary turnover, i.e., species being formed and replacing each other at an unparalleled rate. This result is also in accordance to the observation that South American plant diversity is characterized by a relatively large number of recent, species-rich radiations, for instance in the tropical Andes (Hughes and Eastwood, 2006; Drummond et al., 2012; Madriñán et al., 2013) and Amazonia (Richardson et al., 2001; Erkens et al., 2007). Diversification in the region has been linked to the substantial changes in the landscape in the Neogene (Hoorn et al., 2010; Wesselingh et al., 2010), but several taxa may have an even younger origin in the Quaternary (Rull, 2011; Smith et al., 2014).

### Reliability of results: pushing the limits of biological data

Evolutionary biology and biogeography are now experiencing a tremendous accumulation of data, including molecular sequences, fossils, and species occurrences, with a hitherto unrealized scientific potential. An emerging question, however, is to what extent available data and methods are sufficient to provide us with reliable answers to some of the most fundamental questions in biology. A critical evaluation of the data, methods and assumptions is therefore crucial but often underestimated in evolutionary studies.

Whenever possible, palaeontological data should be studied in conjunction with molecular-based evolutionary analyses (Quental and Marshall, 2010; Fritz et al., 2013; Silvestro et al., 2014). However, our assessment of angiosperm fossils currently available (Figure 1) suggests that data unavailability is a serious issue for angiosperms. The number of angiosperm fossil occurrences publicly available varied considerably among countries and geological periods, with some countries (e.g., USA, Russia) and periods (e.g., the Miocene) being considerably better represented than others. On a continental scale, lack of data is particularly critical for Africa, Southeast Asia and Australasia; but even within relatively well-sampled continents (such as Europe and South America) there are strong regional biases among countries.

Similar to the case of fossil data, there is general skepticism concerning the use of publicly available species occurrences for understanding species distributions, especially from non-verified databases such as GBIF. Distribution data have been shown to contain important taxonomic, temporal and spatial biases (Boakes et al., 2010). The question of whether bioinformatic tools may correctly infer biodiversity patterns despite those biases remains largely unanswered, and will also depend on the scale and taxa in focus—with higher accuracy expected for well-studied taxa and large spatial units. Recent studies suggest that automated data handling procedures are able to yield biologically realistic results, if enough care and appropriate techniques are employed (Zanne et al., 2014; Engemann et al., 2015; Maldonado et al., accepted). In other cases, the manual validation by taxonomists appears crucial, e.g., for the assessment of species' conservation status for the IUCN Red List of Threatened Species (Hjarding et al., 2014).

Our approach of automatically coding species into regions and calculating sampling fractions using GBIF data and polygons is not intended to replace the time-consuming work by taxonomists. However, it constitutes an additional, data-derived and spatially explicit approach that deserves further exploration and validation. Estimating global and regional patterns of species richness and biodiversity remains a notoriously difficult and contentious topic, with no consensus reached (Govaerts, 2001; Crane, 2004; Ungricht, 2004; Wortley and Scotland, 2004; Chapman, 2009; Mora et al., 2011). In addition, there is no general agreement on how to best define, delimit and name biogeographical regions (Kreft and Jetz, 2010; Holt et al., 2013; Vilhena and Antonelli, 2014), with the implication for this study that the world's three tropical regions are differently circumscribed in the literature. Our study suggests that a relatively stable assignment of species to large regions (as in Figure 2) may be attained through simple, automated filtering steps, in which the addition of increasingly restrictive criteria for coding species results in relatively small differences (Figure 3).

The reconstruction of ancestral character states (such as morphology and geographic distribution) along phylogenies is now common practice in evolutionary studies, but only make sense when the traits analyzed are phylogenetically structured—i.e., they are not randomly distributed across the tree. Since we found highly significant clustering of species pertaining to the same geographic assignment in each of the regions defined (Figure 4), we consider that the geographic coding and reconstruction analyses using stochastic mapping are suitable for the goals of this study.

The low taxonomic sampling in the phylogeny (Tables 2, 3) may influence the calculation of range shifts. However, two considerations suggest that this influence is unlikely to significantly affect the general patterns obtained. First, taxonomic sampling varied by only 2% or less among the tropical continents. Second, even at low sampling it should be possible to recover a relatively large proportion of range shifts among the regions outlined. This is because biological sampling is far from being random, with an over-representation of deep nodes that reflect morphological and geographical variations in taxa (Hohna et al., 2011; ter Steege et al., 2011; Cusimano et al., 2012). In other words, even if only a couple of species were sampled from a species-rich but strictly African clade, our analyses should be able to detect when that clade arrived in Africa. Further simulations would be helpful to assess at which sampling levels the calculation of continental-level range shifts stabilize and become fully reliable.

Diversification rates of angiosperms have varied widely among clades (Magallón and Sanderson, 2001) and through time (Silvestro et al., 2015). Inferring the dynamics between speciation and extinction through the Cenozoic for each continent should therefore provide important insights into the evolution of their floras. However, the taxonomic sampling in the angiosperm phylogeny was at or below 10% for all tropical regions (Figure 3, Table 3). Sampling levels already below c. 80% are bound to flaw diversification rate estimates under current methods, often showing slowdowns in net diversification that represent methodological artifacts (Cusimano and Renner, 2010). Expectations on how the missing species are distributed in a phylogeny depending on the sampling scheme may increase the accuracy of diversification analyses (Stadler and Bokma, 2013). However, no method has been developed so far that is capable of confidently dealing with the level of taxonomic sampling observed in the angiosperm phylogeny we used. The MuSSE analyses carried out here can only provide point estimates for the orders surveyed, but should constitute a more powerful approach given the relatively large size of the phylogeny utilized.

### Future prospects: more data, improved methods

The inevitable incompleteness of the fossil record represents a limit to macro-evolutionary analyses that can be carried out using currently available data. However, the development of new methods has shown that even incomplete fossil data can provide essential information in estimating trends of phenotypic evolution (Slater and Harmon, 2013) and species diversification dynamics (Silvestro et al., 2014). Such models should be ideally extended to historical biogeography and might shed new light on the dynamics of migration of lineages through time and among regions. In particular, fossils provide an important resource for improving biogeographic reconstructions, as they provide information on past species ranges and may therefore further refine or validate ancestral range analyses as performed here (Ronquist et al., 2012; Wood et al., 2013; Lawing and Matzke, 2014). Although correct fossil placement on phylogenies can be problematic, their potential in this area is still insufficiently explored (Wood et al., 2013).

Phylogeny-based diversification analyses are powerful complements to palaeontological inferences. However, they still require further development to be confidently used with poorly sampled phylogenies—as is often the case in plants, regardless of geographic region (Figure 3 and Table 3). Until sampling improves to a much higher level (both taxonomically and genetically), or methods currently used successfully with e.g., mammals (Morlon et al., 2011; Stadler, 2011) are adapted and validated for plants, we remain with limited power to assess the dynamics of diversification rates through time and across clades.

## Conclusions

Here we have shown that currently available biological data—including species occurrences and dated phylogenetic trees—hold the potential of providing novel and important insights into large-scale patterns of species diversification and biogeography.

The geographic history of angiosperms involved a large number of range transitions between tropical and non-tropical zones, as well as into and out of the world's three tropical regions. Global climatic changes and major geological events are likely to have influenced some of the observed changes in range shifts, such as the early Eocene climatic conditions and the large geographic reconfigurations in tropical Asia (outlined in Figures 5A,C). However, these are temporal correlations that require further validation. We cannot rule out that some of the fluctuations we observed in the mean rates of range shifts reflect instead the stochastic nature of dispersals and biome shifts, and/or from lack of phylogenetic signal for events that happened tens of millions of years ago.

No significant differences could be found between the speciation and extinction of tropical and non-tropical angiosperms. This result reflects the lack of conclusive evidence on global diversification patterns for different organism groups. Although diversification estimates need to be continuously revalidated with the addition of more genetic and taxonomic data and increasingly robust methods, our results suggest that the latitudinal diversity gradient in angiosperms is not primarily caused by differences in speciation or extinction rates. Longer time for speciation and tropical niche conservatism might therefore constitute better models for explaining tropical angiosperm diversity.

Continental differences in tropical angiosperm diversity show clearer patterns, adding to our knowledge on the global patterns of plant diversity (Kier et al., 2005; Barthlott et al., 2007; Kreft and Jetz, 2007; Kreft et al., 2010; Mutke et al., 2011). The outstanding species richness of angiosperms found today in the Neotropics as compared to tropical Africa and tropical Asia is associated with significantly higher speciation and extinction rates in the Neotropics (Figures 6C,D)—and thereby higher species turnover and shorter average longevity of species. The causes underlying these differences remain elusive, but might be associated with the substantial landscape dynamics that have affected northern South America since the Miocene, among other continent-specific differences such as biome sizes, niche space, and climatic history. Our results also show that Neotropical diversity, once generated *in situ*, was to a large extent “pumped out” of the Neotropics (Figure 5D).

## Data availability

All scripts used in data compilation and cleaning are available upon request.

## Author contributions

AA and AZ conceived this study. AZ, DS, and RS compiled and analyzed the molecular data. BC-M and DS compiled and analyzed the fossil data. All authors interpreted the results and provided input on the manuscript. AA and CDB led the writing with contribution from all authors.

### Conflict of interest statement

The authors declare that the research was conducted in the absence of any commercial or financial relationships that could be construed as a potential conflict of interest.
